# Role of NF-kβ factor Rel2 during *Plasmodium falciparum* and bacterial infection in *Anopheles dirus*

**DOI:** 10.1186/s13071-016-1810-0

**Published:** 2016-09-29

**Authors:** Mohammad Behram Khan, Jonathan Wee Kent Liew, Cherng Shii Leong, Yee-Ling Lau

**Affiliations:** Department of Parasitology, Faculty of Medicine, University of Malaya, Kuala Lumpur, Malaysia

**Keywords:** *Anopheles dirus*, IMD pathway, *Rel2*, *Plasmodium falciparum*, Bacterial infection

## Abstract

**Background:**

*Anopheles* mosquitoes transmit malaria which is one of the world’s most threatening diseases. *Anopheles dirus* (*sensu stricto*) is among the main vectors of malaria in South East Asia. The mosquito innate immune response is the first line of defence against malaria parasites during its development. The immune deficiency (IMD) pathway, a conserved immune signaling pathway, influences anti-*Plasmodium falciparum* activity in *Anopheles gambiae*, *An. stephensi* and *An. albimanus*. The aim of the study was to determine the role of *Rel2*, an IMD pathway-controlled NF-kappaβ transcription factor, in *An. dirus.*

**Methods:**

RACE (Rapid amplification of cDNA ends) was performed on the *Rel2* gene. Double-stranded *Rel2* was constructed and injected into the thorax of female mosquitoes. The injected mosquitoes were fed on a *P. falciparum* gametocyte culture and dissected on day 7–9 post-feeding in order to count the oocysts. A survival analysis was conducted by exposing the dsRNA injected mosquitoes to Gram-positive and Gram-negative bacteria.

**Results:**

This study demonstrated that the *Rel2* gene in *An. dirus* has two isoforms, short length and full length. RNA interference-mediated gene silencing of *Rel2* showed that the latter is involved in protection against *P. falciparum*, Gram-positive bacteria (*Micrococcus luteus*) with Lys-type peptidoglycan and Gram-negative bacteria (*Escherichia coli*) with DAP-type peptidoglycan.

**Conclusion:**

This study suggested that there are similarities in the splicing events and functionality of the *Rel2* gene, between the *Anopheles* species. Among all the important anophelines, the immunity of only a few has been thoroughly investigated. In order to develop novel vector-based control strategies and restrict malaria transmission, the immune pathways of these important vectors should be thoroughly investigated.

**Electronic supplementary material:**

The online version of this article (doi:10.1186/s13071-016-1810-0) contains supplementary material, which is available to authorized users.

## Background

Malaria, caused by the protozoan parasites *Plasmodium* spp., is among the world’s most life threatening infectious diseases. About 70 species of *Anopheles* are capable of transmitting malaria [1]. Nearly 2 billion humans are at risk of contracting malaria in the South East Asia Region (SEAR) [2]. *Anopheles dirus* (*sensu stricto*) (*s.s*) (species A) is an important vector and can be found in Thailand, Myanmar, Cambodia, Vietnam and Laos [3]. The highly anthropophilic nature of *An. dirus A* is also one of the reasons for this species being the main malaria vector in Thailand [3, 4]. A multifaceted approach would be required to curb the disease, especially in light of recent problems that include drug-resistant parasites and insecticide-resistant mosquitoes [5].

Insect immunity exclusively relies on the innate immune response and is devoid of the adaptive immune response. The model organism for the insect immunity, *Drosophila melanogaster*, has provided insights on the two main regulatory pathways, Toll and IMD. In *Drosophila*, the NF-kβ factors associated with the Toll pathway are *Dif* and *Dorsal*, whereas *Relish* (*Rel*) is involved in the IMD pathway [6, 7]. In *An. gambiae, Rel1* is an analogue of *Dif*, and *Rel2* is the orthologue of the *Relish* gene [8, 9]. The most effective anti-*Plasmodium* immune factors, *TEP1*, *APL1*, *LRRD7* and *FBN9*, are all regulated by the NF-kβ *Rel2* transcription factor [10–13]. In *Anopheles*, the *Rel2* gene undergoes alternative splicing to produce a full-length form (*Rel2*-F) and a shorter form (*Rel2*-S). The full-length form consists of the Rel-homology Domain (RHD), carboxyl-terminal ankyrin (ANK) and death domains, whereas the shorter form lacks these inhibitory domains.

The *Drosophila* IMD pathway is activated against Gram-negative bacteria and some Gram-positive bacilli, which contain diaminopimelic acid (DAP-type) in their peptidoglycan (PGN). On the other hand, fungi and Gram-positive bacteria with lysine (Lys-type) in the PGN switch on the Toll pathway [6, 14]. The PGN-recognition proteins (PGRPs) in *An. gambiae* and *Ae. aegypti* activate the IMD pathway, irrespective of the type of PGN present [15] [16].

In this study, we elucidated the role of *Rel2* in *An. dirus* (*s.s*) by using RNA interference (RNAi) during *Plasmodium falciparum* and bacterial infections.

## Methods

### Mosquito rearing

*Anopheles dirus A* (WRAIR2) strain from Malaria Research and Reference Reagent Resource Center (USA) was colonized in the lab using the forced copulation technique. The mosquitoes were maintained at 25 °C, relative humidity of 85 %, with a 12-h light/dark photocycle and fed on a 10 % sucrose solution.

### *Rel2* primer design

A short conserved region of *Rel2* (241 bp) was amplified by designing primers based on the GenBank sequence for *An. quadriannulatus* (Accession No. EU304622.1), *An. coluzzi* (Accession No. KP274426.1), *An. gambiae* M isolate (Accession No. GU990219.1) and *An. merus* (Accession No. KP274430.1). *Rel2* forward and reverse primer sequences: 5′-CAA GTT TCG CTT CCG CTA CCA G-3′ and 5′-ACC CAC ATC CAG ATC GTG-3′.

### Rapid amplification of cDNA ends (RACE)

Total RNA from adult female mosquitoes was extracted using ReliaPrep™ RNA Tissue Miniprep System (Promega, Fitchburg, USA) according to the manufacturer’s instructions. The extracted RNA was used as the template in SMARTer™ RACE cDNA amplification kit (Clonetech, Mountain View, USA) to obtain the full cDNA sequence. The sequence obtained from *Rel2* PCR was utilized in designing primers for RACE. The gene specific primers (GSP) and nested gene specific primers (NGSP) used to amplify the 5′ and 3′ RACE products are shown in Table 1.

**Table 1:** Primers used for rapid amplification of cDNA ends, RT-qPCR and dsRNA

Primer	Sequence 5′-3′
RACE 5′ *Rel2* GSP	CGCTTCTGCGGGTCCACCTGGTACAGGGG
RACE 5′ *Rel2* NGSP	CAGCGAGCCGTGCGTACCGTGCATCTCC
RACE 3′ *Rel2* GSP	GCACGGTACGCACGGCTCGCTGATGGGC
RACE 3′ *Rel2* NGSP	CGCCGGTGAGGCTAAGGTGCGCTGCTCC
RT-qPCR *S7*-F	AGAAGAAGTTCTCCGGTAAG
RT-qPCR *S7*-R	CGGTCTCTTCTGCTTGT
RT-qPCR *Rel2*-F	GGCAGGACAATCTACAAAC
RT-qPCR *Rel2*-R	CTCCAGGATCACGAGATAG
ds*Rel2*-F	CCCTACTGAATTTGGACAGC
ds*Rel2*-R	ATTTGCTGCTGTTGGGACTG
ds*LacZ*-F	CATTACCAGGCCGAAGCAG
ds*LacZ*-R	GCGGCTGATGTTGAACTGGAAG

### RNAi gene silencing

The sequence obtained from RACE was used to construct double-stranded RNA using the T7 Megascript Kit (Ambion, Foster City, USA). A 445 bp *Rel2* region was amplified and the amplicon was ligated downstream of the T7 promoter in the pGEM®-T Vector (Promega) to produce sense and antisense RNA strands. Approximately 50–200 nl of dsRNA (2 μg/μl) was injected into the thorax of anesthetized 2–4 days old female mosquitoes by using InjectMan® NI 2 (Eppendorf, Hamburg, Germany) and FemtoJet® express (Eppendorf) with Femtotips® I (Eppendorf) microcapillaries. The *dsLacZ* (441 bp) was used in the control injection. The primers used for the construction of ds*Rel2* and ds*LacZ* are shown in Table 1.

### Reverse transcription-qPCR

The cDNA was synthesized using the QuantiTect® Reverse Transcription (Qiagen, Hilden, Germany) according to the manufacturer’s instructions. A reverse transcription quantitative PCR was carried out using the iQ™ SYBR® Green Supermix (Bio-Rad, Hercules, USA) on CFX96™ Real-Time System, C1000™ Thermal cycler. The primers are shown in Table 1. All qPCR reactions were performed in triplicates. The melting curves were obtained for each data point to validate the specificity of the PCR reactions. The expression level of *Rel2* in *dsRel2*-treated samples was compared to that of *dsLacZ*-treated samples by normalizing the cDNA levels using the ribosomal protein *S7* gene.

### Gametocyte culture of *P. falciparum* and mosquito infection

Gametocytogenesis in the NF54 strain of *P. falciparum* was induced using an established protocol [17]. On day 16, the culture was pelleted and resuspended in an equal volume of heat-inactivated human serum before transferring to a membrane feeder set at 37 °C, and fed to mosquitoes (injected with dsRNA on day 12 of the *P. falciparum* culture). The fully engorged female mosquitoes were maintained at 25 °C for 7–9 days. Mosquitoes that survived were dissected and the midguts were stained with mercurochrome to check for oocysts. Analysis of the infection prevalence was performed using the Chi-square test whereas for oocyst infection intensity, it was determined by the Mann-Whitney *U* test. The experiment was performed with at least 3 biological replicates and the data were pooled if there was no significant difference between the replicates within the dsRNA treatments.

### Bacterial challenge

*Escherichia coli* and *M. luteus* were cultured to OD_600_ 0.7, pelleted, washed and resuspended in PBS. The final OD_600_ for infection was 0.4 for *E. coli* and 0.1 for *M. luteus*. Four days after the dsRNA injection, mosquitoes were infected with the bacteria by inserting Femtotips® I (Eppendorf) microcapillary in the bacteria culture and poking it in the mosquito’s thorax. The adult mosquitoes that did not recover after 3 h of being exposed to the bacteria were not considered for the analysis. Dead mosquitoes were counted daily and recorded for 10 days. At least three independent experiments were performed, each with at least 25 female mosquitoes per group. The survival analysis was performed using Kaplan-Meier log-rank test.

## Results

### *Rel2* knockdown efficiency

**Fig 1:**
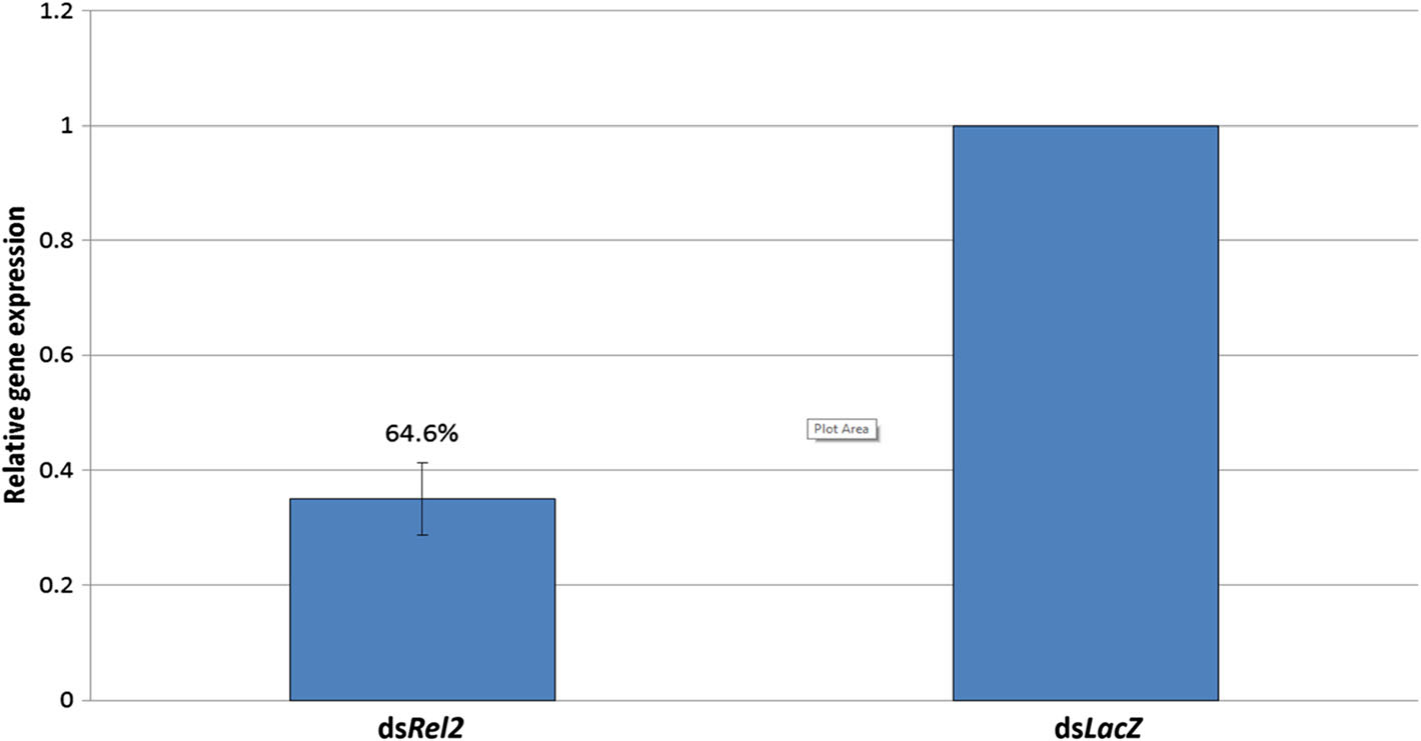
dsRNA mediated silencing efficiency of *Rel2.* The 2^−ΔΔC^_T_ method was utilized to determine the relative gene expression from qPCR data with *S7* as an endogenous reference gene. The silencing efficiency of *Rel2* knockdown was 64.6 ± 6.3 %

Four days after the dsRNA injection, RNA was extracted and converted to cDNA to be used in reverse transcription quantitative PCR. The primers shown in Table 1 were used to determine the *Rel2* silencing efficiency. The 2^−ΔΔC^_T_ method was used to determine the relative gene expression of *Rel2* with *S7* as an endogenous reference gene. At least triplicates were performed in the RT-qPCR reaction. The knockdown efficiency calculated was 64.6 % (Fig. 1).

### Knockdown of *Rel2* increases susceptibility to *P. falciparum* infection in *An. dirus*

In order to investigate the function of *Rel2*, an RNAi mediated gene silencing approach was used. RACE results showed that *Rel2* has two transcripts: a short (*Rel2*-S) and a full-length form (*Rel2*-F). The length of the short form (*Rel2*-S) was around 1.7 kb whereas the full length (*Rel2*-F) covered approximately 3.2 kb. Since dsRNAs act only against mRNAs that contain the corresponding sequence [18], the *dsRel2* construct was designed to silence both forms of the gene by targeting the 5′ region of the short and full length transcripts.

The strain of *P. falciparum* used for infection in this study did not have an unnaturally high infection level in the mosquito [19]. In order to obtain a substantial infection, the *P. falciparum* culture at 0.3 % gametocytemia was fed to the mosquitoes [20]. A higher gametocytemia (≥ 2 %) resulted in infection rates that were undetectable in both groups of dsRNA-treated mosquitoes.

**Fig 2:**
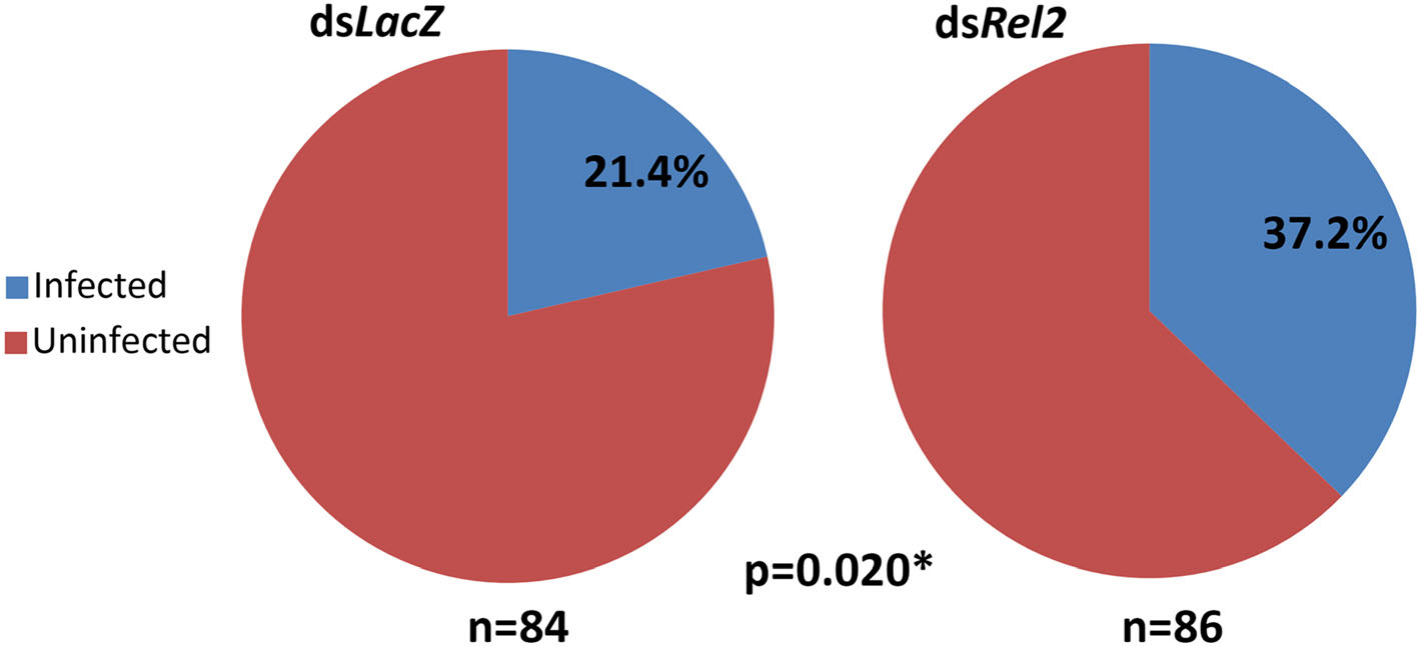
*Anopheles dirus* utilizes *Rel2* against cultured *Plasmodium falciparum*. Silencing of the *Rel2* gene showed significantly higher infection prevalence in *An. dirus* fed on cultured *P. falciparum* gametocytes. The ds*LacZ-*treatedted control had an infection prevalence of 21.4 % whereas the ds*Rel2* injection in the mosquitoes resulted in a significantly higher prevalence of 37.2 % (*χ* ^2^ = 5.39, *df* = 1, *P* = 0.020)

**Fig 3:**
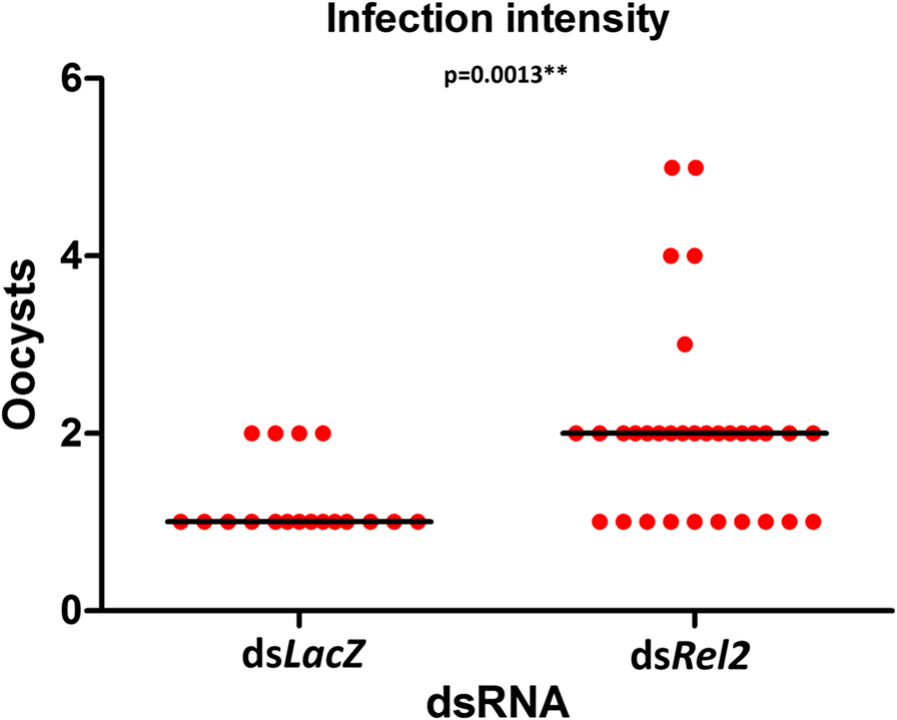
*Rel2* restricts development of *Plasmodium falciparum* in *Anopheles dirus.* Oocyst infection intensity was significantly higher in ds*Rel2-*treated mosquitoes compared to those with ds*LacZ* injected. The dots represent the individual oocyst and the horizontal bars represent the median number of oocysts detected. The P-value was calculated using the Mann-Whitney statistical test. (Mann-Whitney *U* = 144.0, *P* = 0.0013)

Time point gene expression experiments conducted at 2, 12, 24 and 48 h post *P. falciparum* blood-feeding showed that *Rel2* is upregulated the most (1.94-fold) at the 2 h interval (Additional file 1: Figure S1). Silencing the IMD pathway factor, *Rel2*, produced a phenotype that led to a significant increase in infection prevalence as compared to the *dsLacZ*-treated controls (*χ*^2^ = 5.39, *df* = 1, *P* = 0.020; Fig. 2). The *dsLacZ*-treated controls had a median oocyst number of 1, with infection prevalence of 21.4 %, while the *dsRel2*-treated mosquitoes had a 2-fold greater median oocyst number of 2 and an infection prevalence of 37.2 %. Furthermore, the knockdown of *Rel2* also resulted in significantly higher oocyst infection intensity (Mann-Whitney *U* = 144.0, *P* = 0.0013; Fig. 3).

### *Rel2* is required against bacterial infection in *An. dirus*

The potential role of *Rel2* in the defence against bacterial infection in adult mosquitoes was investigated through RNAi analysis. Two to four day-old female mosquitoes were injected with dsRNA and exposed to *E. coli* (Gram-negative) and *M. luteus* (Gram-positive) after 4 days. RT-qPCR was used to confirm the knockdown of the *Rel2* transcript.

**Fig 4:**
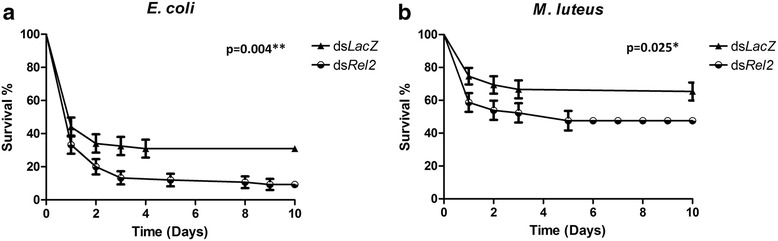
*Rel2* provides protection against Gram-negative and Gram-positive bacteria. Kaplan-Meier survival analysis of dsRNA-treated mosquitoes after being infected with **a** *Escherichia coli* and **b** *Micrococcus luteus*. Each experiment was performed in triplicate with at least 25 mosquitoes per group. The vertical represent standard errors

The preliminary infection experiments suggested the use of O.D_600_ = 0.4 for *E. coli* [21] and O.D_600_ = 0.1 for *M. luteus*. The mosquito survival study was conducted for 10 days after the bacterial infection. *Rel2* knockdown severely compromised the survival of the adult mosquitoes when exposed to *E. coli* (*χ*^2^ = 8.19, *df* = 1, *P* = 0.004; Fig. 4a). A similar trend was observed during the *M. luteus* infection in the *Rel2*-silenced mosquitoes when compared to the *dsLacZ*-treated controls (*χ*^2^ = 5.05, *df* = 1, *P* = 0.025; Fig. 4b).

## Discussion

It is relatively much more convenient to conduct experiments on naturally mating strains compared to colonies maintained through forced copulation. The degree of penetration of the microcapillary in the thorax of the mosquito had an immense effect on the mortality rate, post-injection. Adept and experience in the use of the micromanipulator improves mosquito survival rate to more than approximately 80 %. The limitation during the injection process was the varying volume of dsRNA injected; the volume was estimated by dividing the volume of dsRNA loaded in the microcapillary with the total number of injections possible, at the set parameters.

In the *Drosophila* model system, *Dredd* cleaves the ankyrin repeat region of the *Relish* gene, resulting in the translocation of the active form of *Relish* into the nucleus to activate the IMD pathway [22]. In *Aedes*, the orthologue of *Relish*, *Rel2*, has one additional alternatively spliced transcript than that of *Anopheles*, encoding only the ANK domain [23]. Since *Rel2*-S cannot be targeted specifically without affecting *Rel2*-F, the distinct roles of each are still not evident. However, the *Rel2*-F can be independently silenced to elucidate the function by targeting the region encoding the ankyrin repeats. One study reported *Rel2*-F as having no effect on the prevalence of *P. falciparum* infection [10]. Hemozoin treatment was shown to induce the full length form of *Rel2* and reduce *P. berghei* infection in *An. gambiae* [24]. Garver and colleagues showed that *Rel2*-F had a mild effect on *P. falciparum* oocyst intensity in *An. gambiae*, which was not as potent as the *Rel2*-S and *Rel2-F* combination [25]. Even though the Toll and Jak-Stat pathways are involved in providing protection against *P. berghei*, *P. falciparum* and *P. vivax* [26–29], the IMD pathway has come out as the most effective against human malaria [25]. Our results also demonstrated that *dsRel2*-treated *An. dirus* mosquitoes have a significantly higher *P. falciparum* prevalence and oocyst infection intensity. *Anopheles gambiae* was shown to have an average of 2.2 oocysts per midgut after being infected with *P. falciparum* [30]*.* The knockdown of *Rel2* in the Ngousso mosquito strain showed an increased susceptibility to *P. falciparum* in terms of infection prevalence [10]. Silencing the negative regulator (*Cactus*) of *Rel2* or overexpressing the *Rel2* transcription factor provides nearly complete refractoriness in laboratory reared *An. gambiae*, *An. stephensi* and *An. albimanus* against cultured *P. falciparum* [27]*.* The overexpression of *Rel2* in the midgut tissue of *An. stephensi* following a blood meal also resulted in inefficient invasion of the ookinetes [20]. Silencing *Imd*, *Fadd*, *Dredd* and *Rel2* had a significant effect on the infection intensity, but only the silencing of *Rel2* had a significantly higher infection prevalence [25].

An upper limit for gametocyte density exists, beyond which the infection rates plummet [31, 32]. It seems that the low gametocyte density is an evolutionary mechanism to guarantee fertilization. The high gametocyte densities are extremely rare and probably of little significance epidemiologically. This explains the absence of oocysts when the mosquitoes were fed at a higher gametocytemia.

**Fig 5:**
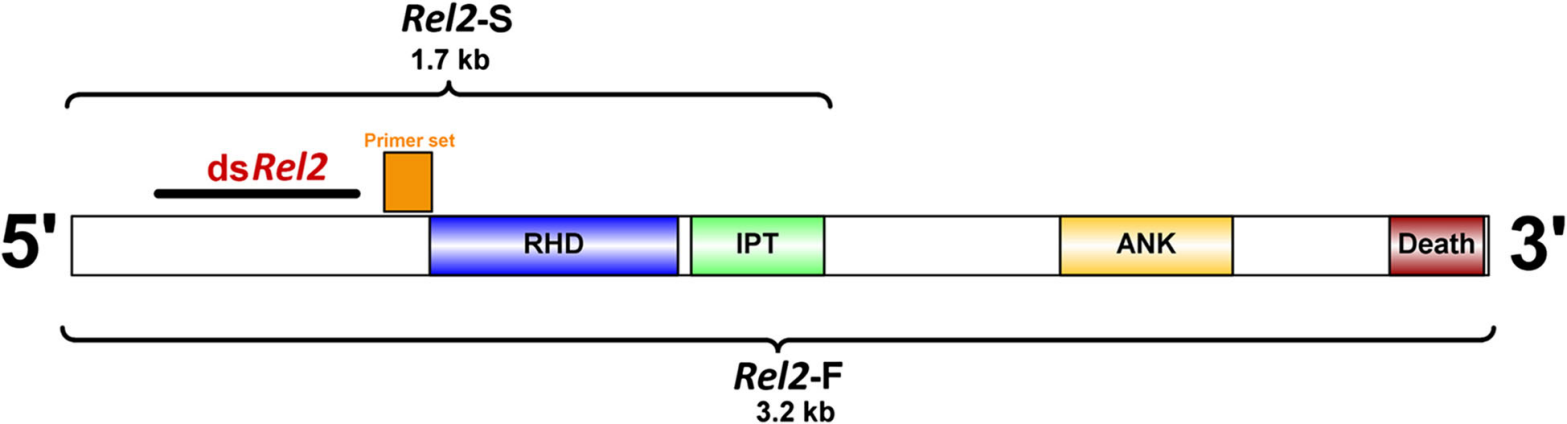
Schematic diagram of *Rel2* and the positions of ds*Rel2* and RT-*Rel2* primers. The ds*Rel2* primers targeted a 445 bp region near the 5′ end of the *Rel2*-S and *Rel2*-F isoforms. The RT-*Rel2* primer set amplified a region of 107 bp, downstream of the ds*Rel2* target region and away from the 3′ end of the gene

The position of the primer plays a pivotal role in detecting the accuracy of silencing in a given sample [33] because some primer sets may be able to bind to the cleaved products and amplify them, resulting in an underestimation of the silencing efficiency [34]. The primers targeting the 3′ end of the mRNA might give false negative results, probably because of the presence of secondary structures or RNA binding proteins [35]. Therefore in this study, the primers were designed away from the 3′ end of the *Rel2* mRNA and downstream of the region targeted by the ds*Rel2* (Fig. 5). However, it is not always possible to cover a specific region of the mRNA because of the thermodynamic limitations posed in primer design for RT-qPCR.

Our results help to explore whether the role of *Rel2* is conserved among different malaria vectors. There may well be differences among the anopheline species in some elements of these downstream mechanisms, but the collective effect results in an anti-*Plasmodium falciparum* immune response; the presence of multiple factors working in tandem would make it highly unlikely for *P. falciparum* to acquire resistance against this pathway [27].

In *Drosophila*, activation of the Toll or the IMD pathway depends on the detection of the PGN on the bacterial surface by the PGRPs. The Toll pathway is responsible when the Lys-type PGN is recognised by the PGRP-SA and PGRP-SD, whereas the IMD pathway is activated upon recognition of the DAP-type PGN by the PGRP-LC. However, the PGRP-LC in *An. gambiae* acts as the main receptor for Lys-type and DAP-type PGN and activates the IMD pathway via translocation of *Rel2*-F and *Rel2*-S into the nucleus, respectively [16]. RNAi-mediated knockdown of *Rel2* in *Ae. aegypti* demonstrated a greater mortality to Gram-negative bacteria *Enterobacter cloacae* and the Gram-positive *Enterococcus faecalis* [15]. Our results showed that *Rel2* provides protection against DAP-type Gram-negative and Lys-type Gram-positive bacteria. Since the AMP genes such as *Defensin*, *Cecropin*, and *Gambicin* can be induced and regulated by both, *Rel1* and *Rel2* [36], it is possible that *Rel1* can contribute to providing protection against certain PGNs, albeit at a lesser extent than *Rel2.* Since the ds*Rel2* in this study targeted the common domains of the *Rel2*-S (short form) and *Rel2*-F (full length), there is a possibility that the *Rel2-*F also has the potential to act against the pathogen/parasite. Therefore, in order to investigate the function of *Rel2*-F, it has to be independently targeted.

## Conclusions

Although all human malaria species of *Plasmodium* are found in SEAR, *P. falciparum* and *P. vivax* are the most prevalent, with the latter species contributing more to the clinical cases currently [37]. However, the biggest threat in this region remains the emergence of *P. falciparum* resistance to artesunate [38]. In SEAR, the number of anopheline species poses a serious problem for malaria control programmes. The results presented in this study explore the role of *Rel2* in an important vector in SEAR and adds to the growing information about mosquito immunity. Evidence from previous studies and this one portrays the significance of *Rel2* against *P. falciparum* and bacterial infection. Nonetheless, *Rel2* has the tendency to affect non-immune genes and the upregulation of the transcription factor may inadvertently have an impact on mosquito fitness and fecundity [39]. Further research has to be conducted to fully comprehend the ramifications of altering the natural gene expression of the mosquito. A thorough investigation of the Jak-STAT, Toll, and IMD pathway in the anopheline species would serve as stepping stone towards generating parasite resistant mosquitoes.

## Additional file

Additional file 1: Figure S1.
*Rel2* gene expression analysis. The mRNA expression level of *Rel2* at different time points after being exposed to *P. falciparum* mature gametocytes. The 2 h interval depicted the highest fold change (1.94-fold) compared to the other time points. The vertical lines represent the standard deviation. (JPG 199 kb)

## Abbreviations

ANK: Ankyrin
ATCC: American type culture collection
dsRNA: Double-stranded RNA
GSP: Gene specific primers
IMD: Immune deficiency
IPT: Immunoglobulin-like, plexins, transcription factors
NGSP: Nested gene specific primers
PGN: Peptidoglycan
PGRPs: PGN-recognition proteins
*Rel*: *Relish*
RHD: Rel-homology domain
RNAi: RNA interference
*s.s*: *sensu stricto*
