# Supplementary figures and images for: Role of NF-kβ factor Rel2 during Plasmodium falciparum and bacterial infection in Anopheles dirus
Source: Parasit Vectors. 2016 Sep 29;9:525. doi: 10.1186/s13071-016-1810-0 (PMC5041562; doi:10.1186/s13071-016-1810-0)

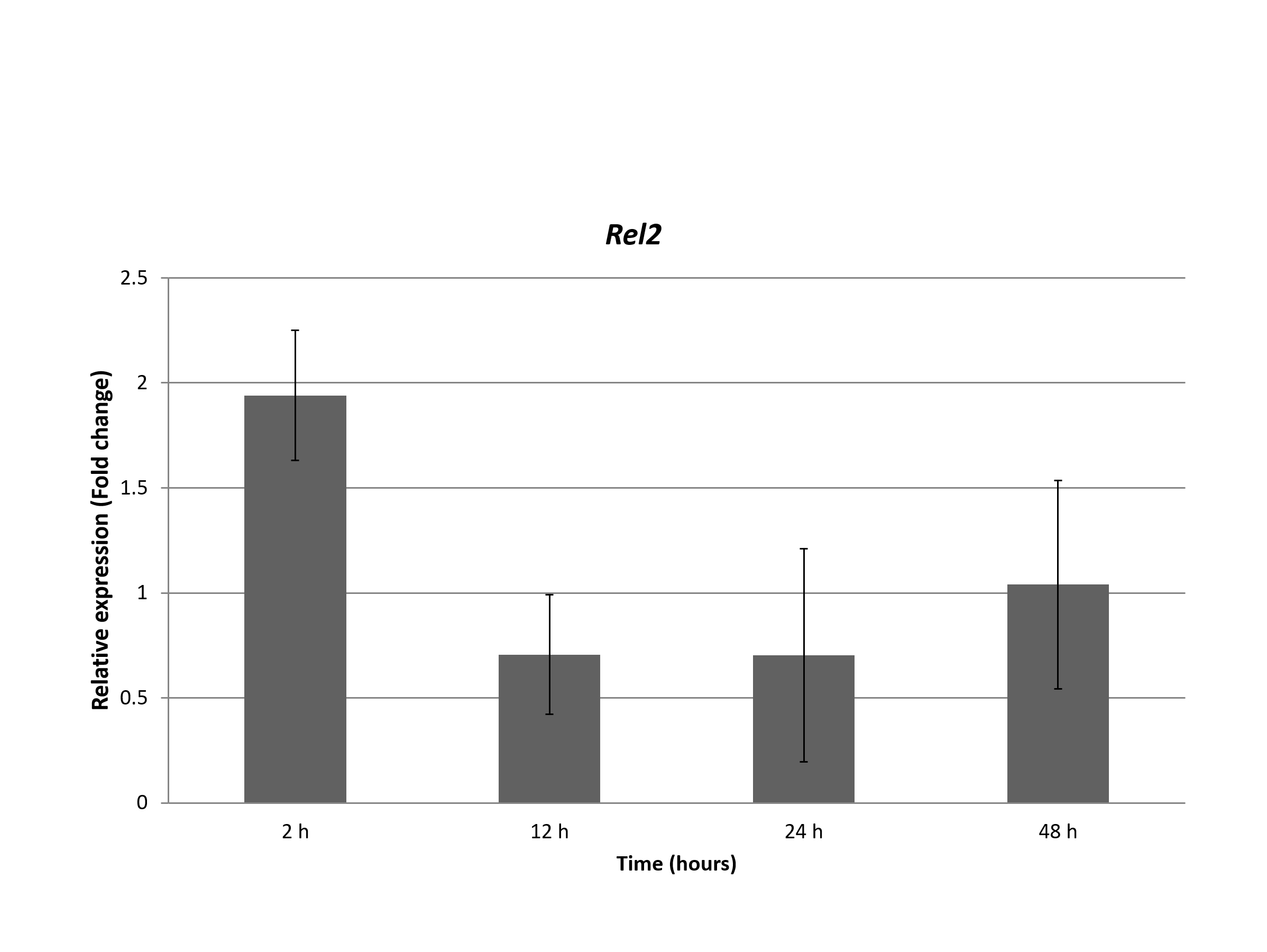

Supplement: Additional file 1: Figure S1. — Rel2 gene expression analysis. The mRNA expression level of Rel2 at different time points after being exposed to P. falciparum mature gametocytes. The 2 h interval depicted the highest fold change (1.94-fold) compared to the other time points. The vertical lines represent the standard deviation. (JPG 199 kb) [file 13071_2016_1810_MOESM1_ESM.jpg]
